# Perioperative Oral Immunonutrient Regulation of Intestinal Barrier and Gut Microbiota in Patients with Gastric Cancer, a Randomized Controlled Clinical Trial

**DOI:** 10.3390/biomedicines13092163

**Published:** 2025-09-05

**Authors:** Zicheng Zheng, Guanmo Liu, Yihua Wang, Jie Li, Chenggang Zhang, Yajun Zhang, Xin Ye, Weiming Kang

**Affiliations:** Department of General Surgery, Peking Union Medical College Hospital, Chinese Academy of Medical Sciences and Peking Union Medical College, Beijing 100005, China

**Keywords:** gut microbiota, intestinal mucosal barrier, enteral nutrition, nutrient–gut microbiota interactions, gastric cancer

## Abstract

**Background and Objectives**: Perioperative enteral and parenteral nutrition have been increasingly used to treat malnutrition in patients with gastric cancer. Immunonutrients have been suggested to reduce postoperative inflammatory responses and enhance immune function compared to conventional nutritional formulas. However, the insufficiency of evidence and unclear specific mechanism limit the recommendation level of immunonutrients in clinical guidelines. This study aimed to investigate the effects of immunonutrients on intestinal barrier function and to explore potential mechanisms through gut microbiota modulation. **Methods**: A total of 58 patients who underwent gastric cancer surgery participated in this randomized controlled trial. The immunonutrients group (*n* = 29) was additionally supplemented with 282 mg of omega-3 fatty acids, 1.2 g of arginine, and 128 mg of nucleotides per 100 kilocalories compared to the standard nutrients group (*n* = 29). Perioperative serum immune, nutritional parameters, and intestinal barrier markers (diamine oxidase, D-lactate, endotoxin) were evaluated. Fecal microbiota structure and functional pathways were analyzed via metagenomic sequencing. **Results**: Postoperative immune and nutritional parameters showed no statistically significant intergroup differences, though mean value curves suggested a protective trend in the immunonutrients group. The immunonutrients group exhibited significantly lower postoperative diamine oxidase (*p* = 0.043) and endotoxin levels (*p* = 0.043), alongside a substantial increase in microbiota α-diversity (*p* = 0.0005). Probiotic genera such as *Akkermansia* (3.26%) and *Bifidobacterium longum* (2.31%) were enriched in the immunonutrients group. Functional pathway analysis suggested that immunonutrients enhanced intestinal barrier protection. **Conclusions**: Immunonutrients may attenuate surgery-induced intestinal barrier damage in gastric cancer patients by modulating gut microbiota diversity, enriching beneficial taxa, and suppressing pathogenic bacteria.

## 1. Introduction

Gastric cancer remains one of the most prevalent malignancies worldwide, with radical surgery serving as the primary treatment modality [1]. Perioperative stress responses, intestinal ischemia–reperfusion injury, and postoperative fasting often lead to impaired intestinal mucosal barrier function, which can trigger increased intestinal permeability, microbial translocation, and systemic inflammatory responses [2,3]. These factors elevate the risks of postoperative infections, multiple organ dysfunction, and other complications. The integrity of the intestinal mucosal barrier relies on a dynamic balance among tight junction proteins, the mucous layer, and commensal gut microbiota [4]. Gut microbiota contributes to barrier maintenance through metabolic products and immune modulation [5]. Malnutrition is highly prevalent among gastric cancer patients, and perioperative enteral and parenteral nutrition therapies have been increasingly adopted in clinical practice [6,7]. Thus, perioperative nutritional support strategies should not only meet energy requirements but also focus on regulating intestinal mucosal barrier function and microbiota homeostasis.

In recent years, immunonutrients—such as arginine, ω-3 polyunsaturated fatty acids, and nucleotides—have garnered attention for their anti-inflammatory, immunomodulatory, and gut-protective potential [8,9]. Studies suggested that immunonutrients may reduce pro-inflammatory cytokine release by inhibiting the nuclear factor-κB (NF-κB) pathway [10,11]. However, existing research predominantly focuses on isolated parameters (e.g., inflammatory markers or nutritional indices), lacking systematic assessments of microbiota structure, functional pathways, and intestinal barrier biomarkers. Furthermore, there are limited studies of high evidence levels such as randomized controlled trials (RCTs) for perioperative immunonutrients, and there is also a lack of research on the intestinal barrier effect of immunonutrients. All these have limited the clinical promotion of immune nutrients in the real world.

To address these gaps, we designed a single-center, randomized, parallel-controlled clinical trial to investigate the effects of perioperative immunonutrients versus standard nutrient supplementation on intestinal mucosal barrier function and gut microbiota in gastric cancer patients. By measuring serum intestinal barrier markers like diamine oxidase, D-lactate, and endotoxin, as well as immune–inflammatory parameters, and by applying metagenomic sequencing to analyze microbiota composition and function, this study aims to address the following scientific questions: (1) Are immunonutrients superior to standard nutrients in improving intestinal mucosal barrier function? (2) Is the efficacy of immunonutrients associated with gut microbiota remodeling and metabolic pathway regulation? (3) How do dynamic changes in microbiota diversity and specific taxa influence clinical outcomes?

## 2. Materials and Methods

### 2.1. Study Design

This single-center, randomized, parallel-controlled clinical trial evaluated the effects of perioperative immunonutrients versus standard nutrient supplementation on intestinal mucosal barrier function and gut microbiota in gastric cancer patients. The study protocol was approved by the Ethics Committee of Peking Union Medical College Hospital (Approval No. K4073) and registered with the Chinese Clinical Trial Registry (Registration No. ChiCTR2400084026). The trial was conducted from June 2023 to December 2024.

### 2.2. Participants

Inclusion criteria included the following: aged 18–80 years, body mass index (BMI) 18.5–28 kg/m^2^, patients undergoing radical gastrectomy, and signing informed consent. Exclusion criteria included the following: inability to receive oral or tube feeding during the perioperative period; severe cardiac, hepatic, or renal dysfunction; pregnancy; lactation; history of radiotherapy, chemotherapy, or blood transfusion within 2 weeks preoperatively; poorly controlled diabetes or allergies; and use of immunomodulators.

### 2.3. Intervention Protocol

Nutritional supplementation was administered in alignment with real-world clinical practice. The experimental group received a formula of immunonutrients, providing 128 mg nucleotides, 1.2 g arginine, and 282 mg ω-3 fatty acids (EPA + DHA) per 100 kcal. The control group received a formula of standard nutrients. Groups were labeled as follows: PUIA referred to the preoperative control group and PUIB referred to the postoperative control group. PUNA represented the preoperative immunonutrients group and PUNB represented the postoperative immunonutrients group.

Preoperative Phase (3 days before surgery):

Daily oral supplementation at 20 kcal/kg/day was provided for 3 days before surgery. Patients were permitted a low energy intake (e.g., water, rice soup), with total energy intake maintained below 40% of the requirements.

Postoperative Phase

Enteral nutrition was gradually reintroduced starting from the 3rd day after surgery, escalated to 20 kcal/kg/day as tolerated, and continued at this dose until the 14th day after surgery. Probiotics, immunomodulators, or other interventions affecting gut microbiota were prohibited throughout the study.

### 2.4. Data Collection

#### 2.4.1. Immune and Nutritional Indicators

Blood samples were collected 3 days preoperatively before nutrient supplementation (baseline) and on the 3rd, 7th, and 14th days following surgery. Immune parameters (white blood cell count, absolute lymphocyte count, CD3+/CD4+/CD8+ T cell count, IgG, hypersensitive C-reactive protein) and nutritional parameters (albumin, prealbumin, total protein, hemoglobin) were analyzed by Automated Hematology Analyzer and Biochemical Analyzer. Among them, blood cell counting is mainly based on flow cytometry, and blood biochemical analysis is mainly based on enzyme-linked immunosorbent assay.

#### 2.4.2. Intestinal Barrier Indicators

Serum intestinal barrier markers (diamine oxidase, D-lactate, endotoxin) were quantified using “diamine oxidase, lactic acid, bacterial endotoxin assay kit” (Product Model: JY-Po-Color DLT Set). The device primarily consists of a base plate and reagent strips. The base plate is equipped with three reaction wells: a diamine oxidase (DAO) reaction well, a D-lactate (DLC) reaction well, and a bacterial endotoxin (BT) reaction well. (a) Diamine Oxidase (DAO) Activity Assay: Diamine oxidase in the blood oxidizes the substrate putrescine to generate H_2_O_2_. In the presence of peroxidase (POD), H_2_O_2_ reacts with the chromo-genic agent to produce a red-colored product. Within the detection range, the color intensity is directly proportional to the diamine oxidase activity in the blood. (b) D-Lactate (DLC) Concentration Assay: D-lactate in the blood, under the action of D-lactate dehydrogenase (D-LDH) and nicotinamide adenine dinucleotide (Coenzyme I, NAD), produces pyruvate and reduced nicotinamide adenine dinucleotide (NADH). In the presence of diaphorase, NADH reduces 2,2′-di-(p-nitrophenyl)-5,5′-diphenyl-3,3′-(3,3′-dimethoxy-4,4′-biphenylene) dite-trazolium chloride (NBT) to form a blue-violet color. Within the detection range, the color intensity is directly proportional to the D-lactate concentration in the blood. (c) Bacterial Endotoxin (BT) Activity Assay: Bacterial endotoxin in the blood hydrolyzes a β-naphthylamide substrate. The resulting substance undergoes an azo coupling reaction with a diazonium salt to produce a red color. The color intensity is directly proportional to the bacterial endotoxin activity. Pipette 20 μL of centrifuged serum into each of the three reaction wells. Allow the reaction to proceed for 15 min. After drying at room temperature, measure the color intensity using a spectrophotometer to obtain the corresponding results [12,13].

#### 2.4.3. Fecal Metagenomic Sequencing

Fecal samples were collected 3 days preoperatively before nutrient supplementation and on the 14th day postoperatively. DNA was extracted directly from fecal samples. Metagenomic sequencing was performed to analyze microbiota composition and functional pathways. A total of 1 μg of genomic DNA per sample was randomly fragmented into ~350 bp fragments using a Covaris ultrasonicator. Libraries were then constructed through end repair, A-tailing, adapter ligation, purification, and PCR amplification. Libraries were preliminarily quantified using Qubit 2.0. Libraries were diluted to 2 ng/μL. Insert size was verified using Agilent 2100 (Agilent Technologies (China) Co., LTD., Beijing, China). Upon confirmation of expected insert size, library effective concentration was accurately quantified using Q-PCR (effective concentration > 3 nM). Qualified libraries were pooled according to effective concentration and target sequencing data volume, followed by PE150 sequencing.

#### 2.4.4. Bioinformatics Analysis

Raw data from the NovaSeq platform were preprocessed using fastp to obtain clean data for downstream analysis. Removal of paired reads if any read contained adapter sequences. Removal of paired reads if >50% of bases in any read had Q ≤ 5. Removal of paired reads if N content in any read exceeded 10%. For samples with host contamination, reads were aligned to host sequences using Bowtie2 (parameters: --end-to-end --sensitive -I 200 -X 400) to filter out host-derived reads [14,15]. Clean data were assembled using MEGAHIT (parameters: --presets meta-large). Resulting scaffolds were split at N-junctions to yield N-free scaftigs [16,17]. ORFs were predicted from scaftigs (≥500 bp) using MetaGeneMark (default parameters; ORFs < 100 nt discarded). Redundancy was removed from ORFs using CD-HIT (parameters: -c 0.95 -G 0 -aS 0.9 -g 1 -d 0) to generate an initial non-redundant gene catalog [18]. Clean data from each sample were aligned to the initial gene catalog using Bowtie2 (parameters: end-to-end, sensitive, -I 200 -X 400) [16]. Genes with ≤2 mapped reads per sample were filtered out to obtain the final gene catalog [19]. Gene abundance per sample was calculated based on mapped read counts and gene length. Basic statistics, core-pan gene analysis, inter-sample correlation, and Venn diagram analysis of gene numbers were performed using the final catalog. Unigenes were aligned against the Micro_NR database (subset of NCBI NR containing Bacteria, Fungi, Archaea, Viruses) using DIAMOND (parameters: blastp -e 1 × 10^−5^) [20]. Taxonomic assignment was determined using the LCA algorithm. Species abundance per sample (at Kingdom/Phylum/Class/Order/Family/Genus/Species levels) was calculated as the sum of abundances of genes annotated to that taxon. For each sequence, hits with e-value ≤ (minimum e-value × 10) were retained [14,21,22]. Gene count per taxon per sample was the number of annotated genes with non-zero abundance. To assess microbial diversity, we computed alpha diversity using the Shannon index, and beta diversity using Bray–Curtis dissimilarity matrices [21,22]. Principal Coordinates Analysis (PCoA) was conducted using the ade4 package, and statistical significance of group-level differences was tested using ANOSIM (Analysis of Similarities) with the vegan package, reporting R and p values [14,23]. Unigenes were aligned against functional databases (CAZy; VFDB) using DIAMOND (parameters: blastp -e 1 × 10^−5^) [21,24]. Differential taxonomic and functional features were analyzed using LEfSe (Linear discriminant analysis Effect Size) with an LDA score threshold of 4, combining Kruskal–Wallis and Wilcoxon rank-sum tests to identify significant biomarkers across groups. All figures showing ordination were supported by corresponding hypothesis testing results (ANOSIM or LEfSe), unless otherwise stated.

### 2.5. Statistical Analysis

Clinical and laboratory data were analyzed using repeated-measures ANOVA in SPSS 26.0 [25]. Sphericity was tested via Mauchly’s test: if violated (*p* < 0.05) and ε > 0.75, Huynh–Feldt correction was applied; if ε ≤ 0.75, Greenhouse–Geisser correction was used; if sphericity was assumed (*p* > 0.05), unadjusted results were reported [25]. Fecal metagenomic sequencing data were processed using R software (v 2.15.3).

### 2.6. Sample Size and Ethics

**Figure 1:**
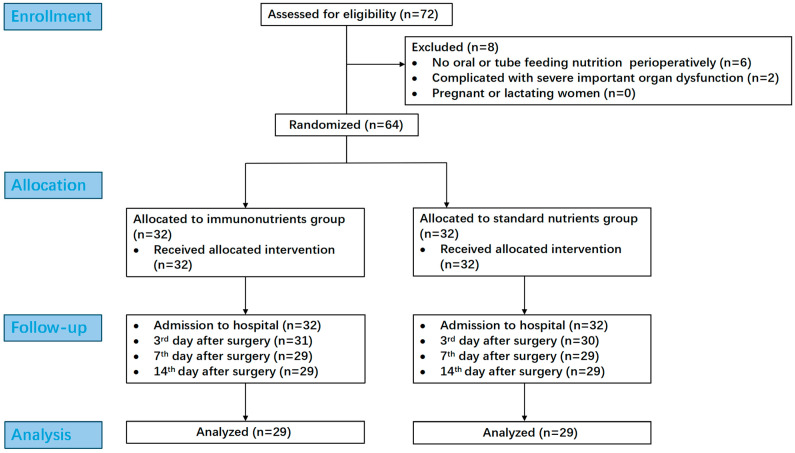
Flowchart.

A total of 72 patients were enrolled, with 8 excluded and 6 incomplete follow-ups (due to nutrient intolerance or postoperative complications), leaving 58 participants (29 patients per group) for final analysis. All procedures adhered to the Declaration of Helsinki. The flowchart is shown in Figure 1.

## 3. Results

### 3.1. Descriptive Analysis

Baseline characteristics, including age, gender, BMI, AJCC stage, and surgical related parameters, showed no significant differences between the control and experimental groups (all *p* > 0.05), confirming balanced comparability. Critically, there was no statistically significant difference in the incidence of postoperative complications between the two groups (Table 1).

### 3.2. Perioperative Immune and Nutrition Parameter Changes

**Figure 2:**
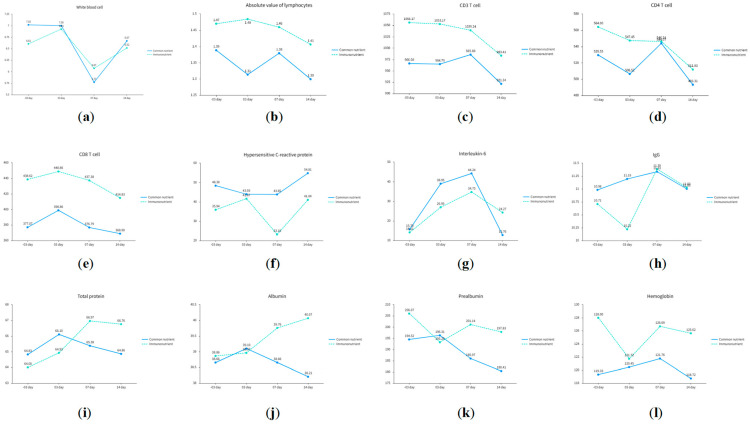
Variation curves of immune and nutrition indicators. This figure illustrates the results of repeated-measures ANOVA for longitudinal changes in immune, inflammatory, and nutrition parameters between the immunonutrients group and standard nutrients group, presented as line charts. (**a**–**e**) display immune cell indicators: white blood cells (WBC, ×10^9^/L), absolute lymphocyte count (ALC, ×10^9^/L), CD3^+^ T cells (cells/μL), CD4^+^ T cells (cells/μL), and CD8^+^ T cells (cells/μL). (**f**–**h**) show inflammatory markers: C-reactive protein (CRP, mg/L), interleukin-6 (IL-6, pg/mL), and immunoglobulin G (IgG, g/L). (**i**–**l**) represent nutrition parameters: total protein (g/L), albumin (g/L), prealbumin (mg/L), and hemoglobin (g/L). Data points indicate group-specific mean values at different time points.

Repeated-measures ANOVA revealed no statistically significant intergroup differences in immune or nutrition parameters (Table 2). However, graphical trends suggested distinct patterns (Figure 2). The experimental group exhibited higher mean levels of functional inflammatory cells (e.g., CD3+, CD4+ T cells), implying enhanced immune responsiveness. For inflammatory markers, lower mean levels of pro-inflammatory cytokines (e.g., hypersensitive C-reactive protein) in the experimental group indicated more stable systemic inflammation. As for nutrition parameters, the experimental group demonstrated gradual postoperative recovery in albumin and total protein levels, while the control group showed declines, suggesting superior nutritional recovery with immunonutrient supplementation.

### 3.3. Perioperative Intestinal Barrier Marker Changes

**Figure 3:**
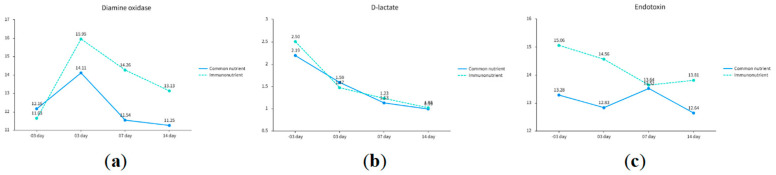
Curve of changes in intestinal barrier indicators. This figure presents the results of repeated-measures ANOVA for longitudinal variations in intestinal barrier biomarkers between the immunonutrients group and standard nutrients group, depicted as line charts. (**a**) shows diamine oxidase (U/mL), (**b**) displays D-lactate (mg/L), and (**c**) illustrates endotoxin (EU/mL). Data points represent group-specific mean values across time points.

Significant time and group effects were observed for diamine oxidase (F = 11.364, *p* < 0.001; group effect F = 2.770, *p* = 0.043) and endotoxin (F = 2.844, *p* = 0.039; group effect F = 2.772, *p* = 0.043). The experimental group maintained stable or reduced diamine oxidase and endotoxin levels postoperatively, consistent with preserved mucosal integrity and attenuated bacterial translocation. In contrast, D-lactate levels showed significant time effects (F = 20.263, *p* < 0.001) but no intergroup differences (*p* = 0.541), indicating comparable trends in intestinal permeability (Table 3) (Figure 3).

### 3.4. Gene Differential Analysis

Some enrolled patients failed to provide fecal samples of the 14th day after surgery due to irregular bowel movements, constipation, or absence of stool caused by a liquid diet. Complete preoperative and postoperative fecal samples were obtained from 20 patients in the experimental group and 20 in the control group. Metagenomic sequencing revealed potential differences in genomic complexity between the PUIA and PUIB groups based on non-redundant gene counts. Venn diagrams indicated that while most genes were shared across groups, hundreds of thousands of gene-level differences existed between them (Supplementary Figure S1).

#### 3.4.1. Phylum-Level Differences

**Figure 4:**
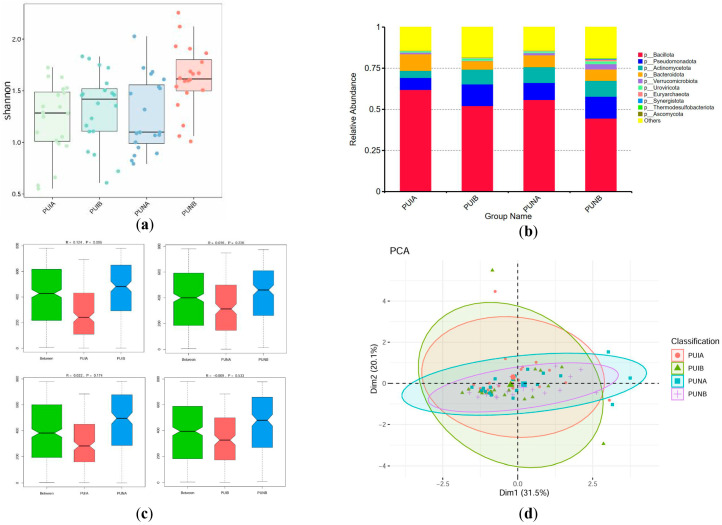
Characteristics of the microbiota at the phylum level. (**a**) Alpha diversity of fecal microbiota assessed by Shannon index. (**b**) Relative abundance of bacterial phyla across groups. Dominant phyla (e.g., *Bacillota*, *Pseudomonadota*) are color-coded. The differences that do not rank high are marked in yellow as “Other”. (**c**) ANOSIM analysis results among each group. The R value reflects the relationship of differences between and within groups (a positive value indicates that the difference between groups is greater than that within groups, while a negative value indicates the opposite), and the *p* value reflects whether there is a statistical difference. (**d**) PCoA analysis based on principal coordinates.

Shannon index analysis at the phylum level demonstrated significantly superior α-diversity in the PUNB group compared to other groups (*p* < 0.05). Specifically, α-diversity in PUNB was 18 units higher than in PUIB (standard nutrients postoperative, *p* = 0.0098, 95% CI: −31.54 to −4.46) and 22.45 units higher than in PUNA (*p* = 0.0015, 95% CI: −35.99 to −8.91), highlighting the unique benefits of immunonutrient supplementation. While *Bacillota* dominated across all groups, PUNB exhibited the lowest abundance of this phylum (44.56% vs. 61.95% in PUIA), suggesting a shift toward a more balanced microbiota. Postoperative groups (PUIB and PUNB) showed elevated *Pseudomonadota* (13.14% in PUIB) and *Actinomycetota* (8.99% in PUIB), but PUNB uniquely displayed the highest proportion of unclassified phyla (“Others”: 18.94%), reflecting enhanced ecological complexity and resilience, likely driven by immunonutrient-induced microbial modulation. ANOSIM analysis confirmed significant divergence between PUIA and PUIB (R = 0.124, *p* = 0.006 for PUIA-PUIB vs. nonsignificant PUNA-PUNB differences). PCoA plots further illustrated that PUNB samples overlapped with preoperative PUNA, indicating stable microbiota preservation in the immunonutrients group despite surgical stress, whereas PUIB clustered distinctly due to pathogenic phylum enrichment (Figure 4).

#### 3.4.2. Genus-Level Differences

**Figure 5:**
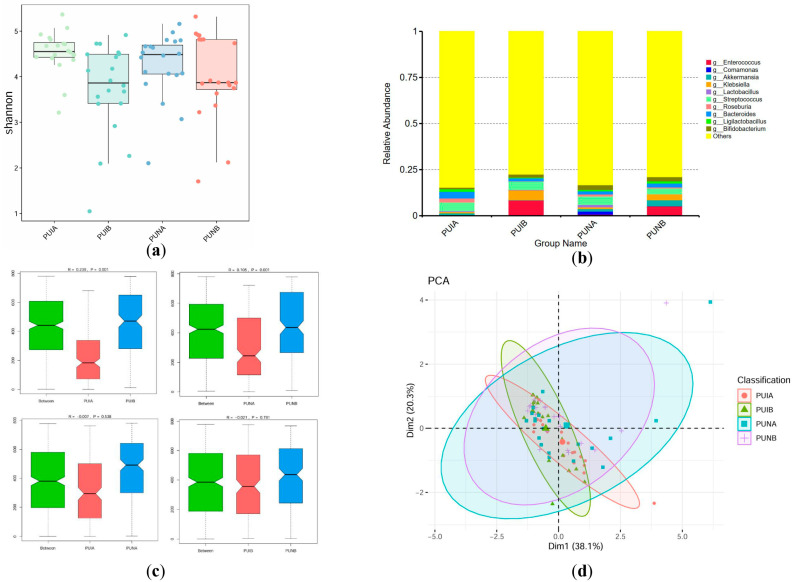
Characteristics of the microbiota at the genus level. (**a**) Alpha diversity of fecal microbiota assessed by the Shannon index. (**b**) Relative abundance of bacterial genera across groups. Dominant genera (e.g., Akkermansia, *Bifidobacterium*, *Enterococcus*) are color-coded. The differences that do not rank high are marked in yellow as “Other”. (**c**) ANOSIM analysis results among each group. The R value reflects the relationship of differences between and within groups (a positive value indicates that the difference between groups is greater than that within groups, while a negative value indicates the opposite), and the *p* value reflects whether there is a statistical difference. (**d**) PCoA analysis based on principal coordinates.

The Shannon index of the PUNB group at the genus level was not significantly different from that of the other groups. (vs. PUIB, *p* = 0.1834, 95% CI: −23.59 to 4.59; vs. PUNA, *p* = 0.5783, 95% CI: −10.14 to 18.04). PUNB demonstrated marked enrichment of beneficial genera, including *Akkermansia* (3.26%, 14-fold higher than PUIB) and *Bifidobacterium* (2.31%), which are critical for mucin degradation, short-chain fatty acid production, and immune regulation. In contrast, PUIB (standard nutrients postoperative) showed proliferation of opportunistic pathogens such as *Enterococcus* (8.40%) and *Klebsiella* (5.25%), genera associated with inflammation and barrier disruption. Strikingly, PUNB maintained lower levels of these pathogens (*Enterococcus*: 5.27%; *Klebsiella*: 3.17%) compared to PUIB, underscoring immunonutrients’ dual role in promoting probiotics and suppressing dysbiosis. ANOSIM analysis showed that there were significant differences in the microbiota before and after the operation at the genus level (R = 0.239, *p* = 0.001 for PUIA-PUIB; R = 0.105, *p* = 0.001 for PUNA-PUNB). PUNB samples diverged toward *Akkermansia*-dominant profiles, aligning with their role in barrier integrity, while PUIB clustered with *Enterococcus*-driven dysbiosis. This contrast emphasizes immunonutrients’ efficacy in reshaping genus-level dynamics toward a protective phenotype (Figure 5).

#### 3.4.3. Species-Level Differences

**Figure 6:**
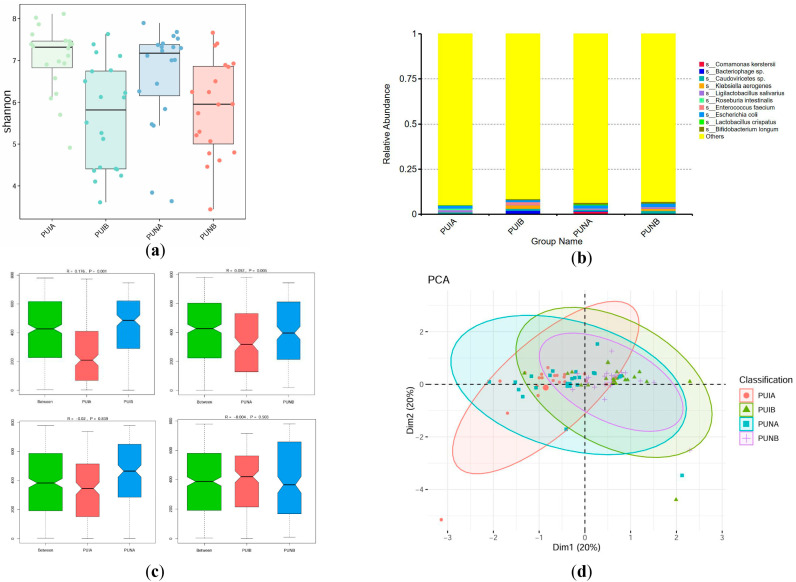
Characteristics of the microbiota at the species level. (**a**) Alpha diversity of fecal microbiota assessed by the Shannon index. (**b**) Relative abundance of bacterial species across groups. Dominant species (e.g., Bifidobacterium *longum*, *Klebsiella aerogenes*) are color-coded. The differences that do not rank high are marked in yellow as “Other”. (**c**) ANOSIM analysis results among each group. The R value reflects the relationship of differences between and within groups (a positive value indicates that the difference between groups is greater than that within groups, while a negative value indicates the opposite), and the *p* value reflects whether there is a statistical difference. (**d**) PCoA analysis based on principal coordinates.

At the species level, the Shannon index of the PUNB group was not significantly different from that of the PUIB group (*p* = 0.6181, 95% CI: −16.43 to 9.83), but significantly different from that of the PUNA group. (*p* = 0.0156, 95% CI: 3.17 to 29.42). PUNB exhibited enrichment of probiotic taxa such as *Bifidobacterium longum* (0.98%, 1.8-fold higher than PUIB) alongside reduced pathogenic species (*Klebsiella aerogenes*: 1.20% vs. 1.94% in PUIB). Although *Escherichia coli* persisted in PUNB (1.83%), its abundance remained lower than typical dysbiotic states. Notably, PUNB showed enhanced bacteriophage activity (0.38% vs. 2.05% in PUIB), potentially contributing to pathogen suppression. ANOSIM analysis showed that there were significant differences in the microbiota before and after the operation at the genus level (R = 0.176, *p* = 0.001 for PUIA-PUIB; R = 0.092, *p* = 0.005 for PUNA-PUNB). PCoA revealed PUNB’s shift toward negative Axis1 values (e.g., PUNB16: Axis1 = −51.22), driven by *Akkermansia* and *Bifidobacterium longum* enrichment, whereas PUIB clustered in pathogen-positive regions. These findings conclusively demonstrate that immunonutrients reprogram species-level interactions to favor a protective, anti-inflammatory gut environment (Figure 6).

### 3.5. Differential Gene Function Analysis

LEfSe analysis revealed significant differences in microbiota composition and functional pathways between PUIB and PUIA. PUIB was enriched with opportunistic pathogens such as *Enterococcus* (genus), *Actinomycetales* (order), and species including *Enterococcus faecalis* and *Bifidobacterium pseudocatenulatum*, which are associated with intestinal inflammation, opportunistic infections, and dysbiosis. In contrast, PUIA exhibited dominance of probiotic taxa such as *Akkermansia* (genus), *Faecaliberium prausnitzii* (species), and *Roseburia* (genus), which enhance barrier function via mucin degradation, butyrate production, and immune regulation. Notably, *Akkermansia muciniphila* and *Roseburia intestinalis* in PUIA were linked to anti-inflammatory effects through aryl hydrocarbon receptor (AhR) activation and regulatory T cell (Treg) differentiation.

**Figure 7:**
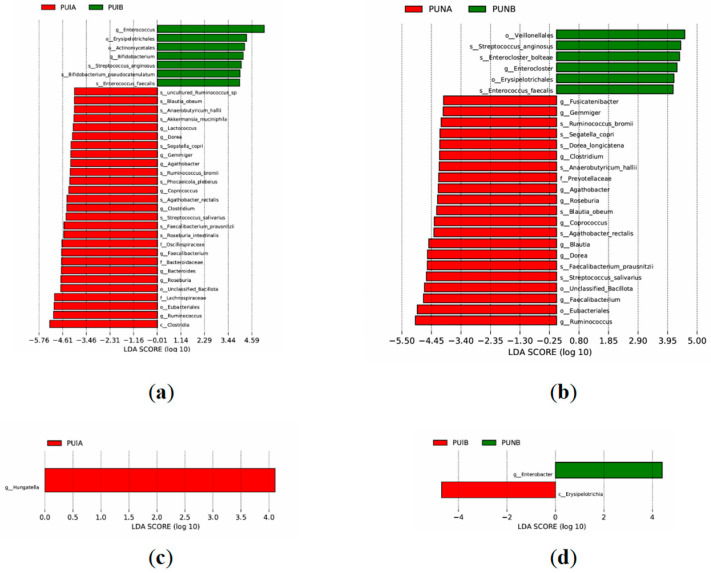
LEfSe analysis of differential bacterial taxa between groups. (**a**) PUIA (preoperative control) vs. PUIB (postoperative control). (**b**) PUNA (preoperative immunonutrients) vs. PUNB (postoperative immunonutrients). (**c**) PUIA (preoperative control) vs. PUIB (postoperative control). (**d**) PUIB (postoperative control) vs. PUNB (postoperative immunonutrients).

PUNA and PUNB comparisons demonstrated distinct functional shifts. PUNB showed enrichment of *Enterococcus faecalis*, *Erysipelotrichales* (order), and pathogenic species like *Enterocloster bolteae* and *Streptococcus anginosus*, suggesting residual dysbiosis risks. However, PUNA maintained higher abundances of *Prevotellaceae* (family) and unclassified *Bacillota* (order), which support fiber metabolism and mucosal protection. Postoperative comparisons (PUNB vs. PUIB) highlighted immunonutrient-specific benefits: PUNB had reduced *Enterobacter* (genus, containing opportunistic pathogens like *Klebsiella aerogenes*), whereas PUIB was enriched with *Erysipelotrichia* (class), linked to metabolic dysfunction and barrier impairment (Figure 7).

Comparison of PUIA and PUIB revealed distinct virulence factor profiles. PUIB showed significant enrichment of virulence genes, including streptolysin O (VF0353) and Esp surface protein (VF0456), which promote cytolysis and biofilm formation. PUIA’s virulence profile was characterized by context-dependent activation (e.g., triggered by mucosal injury), reflecting baseline microbiota–pathogen interactions in the preoperative state.

CAZy annotation highlighted divergent carbohydrate metabolism strategies between PUIA and PUIB. PUIA enriched glycoside hydrolase families (GH2, GH3, GH43) and carbohydrate-binding modules (CBM48, CBM26), which facilitate dietary fiber degradation and short-chain fatty acid (SCFA) production. In contrast, PUIB dominated GH1, GH4, and GH73 families, which degrade host glycoproteins/mucins and remodel pathogenic cell walls, exacerbating mucosal damage and inflammation (Supplementary Figure S2).

These findings suggest that postoperative microbiota in PUIB exhibit enhanced pathogenic potential through virulence factor activation and mucin-degrading enzyme expression, whereas preoperative microbiota (PUIA) prioritize fiber metabolism and barrier-supportive functions.

## 4. Discussion

This study evaluated the regulatory effects of perioperative immunonutrient supplementation on intestinal mucosal barrier function and gut microbiota in gastric cancer patients through a randomized controlled trial. The results demonstrated that the immunonutrients group exhibited significantly lower postoperative levels of diamine oxidase and bacterial endotoxin compared to the standard nutrients group (both *p* < 0.05), alongside significant improvements in microbiota diversity, probiotic abundance, and anti-inflammatory metabolic pathways. The following sections discuss these findings in depth, integrating insights from the existing literature.

Diamine oxidase, an enzyme located in the mitochondria of intestinal epithelial cells, directly reflects physical damage to the intestinal mucosal barrier when its plasma levels are elevated [26]. This study demonstrated that the immunonutrients group exhibited significantly lower postoperative diamine oxidase levels compared to the control group (*p* < 0.05), suggesting that immunonutrients may mitigate surgical stress-induced mucosal damage through multiple pathways. Previous studies indicate that n-3 polyunsaturated fatty acids (e.g., eicosapentaenoic acid [EPA] and docosahexaenoic acid [DHA]), core components of immunonutrients, competitively inhibit arachidonic acid metabolism, thereby reducing the production of pro-inflammatory mediators such as prostaglandin E2 (PGE2) and leukotriene B4 (LTB4) [27,28,29,30,31]. Concurrently, they activate peroxisome proliferator-activated receptor gamma (PPAR-γ), suppress nuclear factor-kappa B (NF-κB) signaling pathway activation, and downregulate the expression of pro-inflammatory cytokines like interleukin-6 (IL-6) and tumor necrosis factor-alpha (TNF-α) [10,11,32,33]. This mechanism has been validated in animal models: EPA/DHA supplementation significantly reduced diamine oxidase levels and decreased apoptotic cell ratios in the intestinal mucosa of mice with ischemia–reperfusion injury [34,35]. The concurrent decline in endotoxin (lipopolysaccharide, LPS) levels (*p* < 0.05) further corroborates the barrier-protective effects of immunonutrients. Arginine in immunonutrients may improve intestinal microcirculation through nitric oxide (NO)-dependent vasodilation, alleviating postoperative mucosal ischemia [36,37]. Nucleotides (e.g., RNA, nucleosides) serve as substrates for rapid regeneration of intestinal epithelial cells, promoting villus repair. The synergistic effects of these components likely form a multi-layered protective network for the intestinal barrier [38,39].

The immunonutrients group appeared to exhibit significantly higher gut microbiota α-diversity (Shannon index) compared to the control group, with a core microbiota structure potentially characterized by probiotic dominance and pathogen suppression. Specifically, the abundance of *Akkermansia* in the PUNB group reached 3.26%, suggesting a nearly 14-fold increase over the PUIB group (0.23%). *Akkermansia* may degrade mucin MUC2 in the intestinal mucus layer through the secretion of mucinases (e.g., Amuc_1100 protein), possibly generating short-chain fatty acids (SCFAs) and oligosaccharides [40,41,42]. This process could stimulate goblet cell proliferation to maintain mucus layer thickness while potentially activating the aryl hydrocarbon receptor (AhR) pathway to promote regulatory T cell (Treg) differentiation, thereby possibly suppressing Th17-mediated excessive inflammatory responses [43,44]. Additionally, the abundance of *Bifidobacterium longum* in the PUNB group (0.98%) was observed to be significantly higher than in the PUIB group (0.55%). *Bifidobacterium longum* might competitively inhibit pathogen adhesion (e.g., Enterococcus) via extracellular polysaccharide (EPS) secretion and could metabolize dietary fiber to produce acetate, potentially lowering intestinal pH and further inhibiting the growth of opportunistic pathogens [45,46]. *Bifidobacterium* and its secreted high-concentration EPS can significantly restore the LPS-disrupted expression of protein Zonulin (ZO-1), thereby repairing impaired intestinal epithelial tight junctions. Furthermore, they inhibit the key pro-inflammatory signaling pathway (TLR-4/NF-κB). This dual action exerts protective effects on the intestinal barrier and modulates the inflammatory response [47]. Furthermore, *Bifidobacterium* and *Akkermansia* produce SCFAs, vitamins, and several small molecules, which contribute to the structural immunity of the intestinal epithelial barrier [48,49]. The enrichment of *Verrucomicrobiota* (3.26%) and *Euryarchaeota* (0.97%) in the PUNB group could hold significant ecological implications. Members of *Verrucomicrobiota* (e.g., *Ruminococcus gnavus*) might ferment dietary fiber to produce succinate, possibly modulating intestinal redox balance [50], while methanogens within *Euryarchaeota* (e.g., *Methanobrevibacter smithii*) may enhance complex carbohydrate fermentation efficiency by consuming hydrogen, indirectly boosting SCFA production [51]. This “metabolic cross-feeding” might optimize intestinal energy supply and contribute to maintaining epithelial homeostasis [52]. Immunonutrients improving intestinal barrier protection and immune regulation may be the result of multiple factors. The key associated signaling pathways remain to be further studied.

In contrast, the microbiota profile of the control group exhibited trends of pathogenic invasion and metabolic dysregulation. The enrichment of *Klebsiella aerogenes* (1.94%) and *Enterococcus faecium* (1.38%) was closely associated with postoperative intestinal microenvironmental disturbances. *Klebsiella aerogenes* evades host immune clearance through capsular polysaccharides (e.g., K antigen) and releases lipopolysaccharide to activate the Toll-like receptor 4 (TLR4)–MyD88-NF-κB pathway, triggering systemic inflammatory responses [53,54]. VFDB annotation further revealed significant enrichment of virulence factors in the PUIB group, including streptolysin O (VF0353) and enterococcal surface protein Esp (VF0456). Streptolysin O lyses host cells by forming transmembrane pores, while Esp enhances pathogen antibiotic resistance by promoting biofilm formation [55,56]. Additionally, CAZy functional analysis indicated heightened activity of GH73 (peptidoglycan hydrolases) and GH1 (β-glucosidases) in the PUIB group, suggesting predatory exploitation of host glycoproteins. This activity may compromise mucus layer integrity, creating a metabolic niche conducive to pathogenic invasion [57,58]. Furthermore, PUNB maintained lower levels of these pathogens (*Enterococcus*: 5.27%; *Klebsiella*: 3.17%) compared to PUIB. The dysbiosis, which includes the abundances of *Klebsiella*, *Parvimonas*, and *Clostridium* increase and those of *Bifidobacterium* and *Lactobacillus* decrease, may promote the formation and recurrence of colorectal polyps [59]. Similar microbial changes have also been observed in colorectal cancer, inflammatory bowel disease, autism spectrum disorder, and metabolic syndrome [60]. Regulating the gut microbiota, particularly through the observed decreases in pathogenic bacterial species and the enhancement of beneficial species, may help promote gut health and have significant implications for systemic inflammation [61,62]. Another study also showed that bacteria including *Klebsiella*, *Ruminococcus,* and *Peptococcus* were abundant in patients with gastric cancer who developed postoperative delirium. It also indicated that the gut microbiota might influence postoperative delirium by altering the tolerance to oxidative stress [63]. Xiao et al. also suggested that patients who had *Klebsiella* detected in bacterial cultures after radical gastrectomy tended to develop intra-abdominal infection [64].

This study has several limitations. Although PUNB showed significant improvements in microbiota diversity (24.75 increase in Shannon index, *p* = 0.0005) and probiotic abundance (e.g., Akkermansia and Bifidobacterium longum), no statistically significant difference was observed in short-term postoperative complication rates between the immunonutrients group (17.24%) and the control group (20.69%) (*p* = 0.738). This discrepancy may arise from the following mechanisms and limitations. During the 14-day postoperative follow-up period, microbiota remodeling may not have fully modulated the host immune–metabolic network. For example, the enrichment of *Verrucomicrobiota* (3.26%) and *Euryarchaeota* (0.97%) in the PUNB suggests potential roles of methane metabolism and acidic microenvironment modulation, but such metabolic adjustments likely require longer durations (e.g., 3–12 weeks) to significantly influence mucosal repair or systemic inflammation markers [65,66]. Moreover, early postoperative complications (e.g., anastomotic leakage) are primarily caused by direct surgical trauma, whereas microbiota-mediated barrier protection may play a more critical role in medium- to long-term outcomes (e.g., infectious complications). Additionally, this study did not conduct an analysis of fecal metabolites, and further follow-up research is needed to improve it. The current sample size (*n* = 29 per group) only provided sufficient power to detect intergroup differences in complication rates ≥25% (α = 0.05, β = 0.8), yet the observed difference was 3.45%. This suggests that expanding the sample size to over 500 cases may be necessary to validate subtle but clinically meaningful trends, though such recruitment is currently constrained by time and economic costs. Furthermore, the small sample size limited the statistical power of subgroup analyses (e.g., stratification by TNM stage), potentially obscuring potential benefits in high-risk populations (e.g., stage III patients).

## 5. Conclusions

Perioperative supplementation with immunonutrients significantly reduced postoperative diamine oxidase and endotoxin levels in gastric cancer patients, indicating the effective protection of intestinal mucosal barrier function by mitigating mucosal damage and suppressing endotoxin release. The PUNB exhibited significantly higher gut microbiota α-diversity compared to the PUIB, with increased abundances of probiotics such as *Akkermansia* and *Bifidobacterium longum*, and decreased abundances of opportunistic pathogens like Enterococcus and Klebsiella. These findings further support the role of immunonutrients in enhancing intestinal barrier integrity and fostering an anti-inflammatory microenvironment. Although no statistically significant differences were observed in postoperative immune/nutritional parameters or complication rates between the immunonutrients and control groups, trends favoring the immunonutrients group suggest potential benefits. The lack of significance may stem from the small sample size, short follow-up duration, or individual heterogeneity. Future studies should expand sample sizes, extend follow-up periods, and integrate multi-omics data to validate the long-term clinical impact of immunonutrient interventions.

## Figures and Tables

**Table 1:** Comparison of basic clinical characteristics between two groups.

Variables	Groups		*p*
	Control Group (*n* = 29)	Experimental Group (*n* = 29)	
Age (years)	59.207 ± 10.311	57.828 ± 12.795	0.653
Gender, n (%) Male	21 (72.41)	19 (65.52)	0.570
Female	8 (27.59)	10 (34.48)	
BMI (kg/m^2^)	23.254 ± 3.207	23.345 ± 3.434	0.918
Hospitalization time (days) Surgical procedure, n (%)	16.414 ± 10.995	13.448 ± 2.585	0.167
Total gastrectomy	12 (41.38)	9 (31.03)	0.644
Distal gastrectomy	1 (3.45)	2 (6.90)	
Proximal gastrectomy	16 (55.17)	18 (62.07)	
Neoadjuvant chemotherapy, n (%) Yes No	10 (34.48) 19 (65.52)	11 (37.93) 18 (62.07)	0.785
Operation time (hours)	3.224 ± 0.867	3.103 ± 0.763	0.576
AJCC stage I	13 (44.83)	12 (41.38)	0.157
II	10 (34.48)	5 (17.24)	
III	6 (20.69)	12 (41.38)	
Postoperative complication, n (%) Yes No	23(79.31) 6(20.69)	24 (82.76) 5 (17.24)	0.738

Abbreviations: AJCC, American Joint Committee on Cancer.

**Table 2:** Comparison of perioperative immune and nutrition indicators between two groups and different times.

Variables	Statistical Indicators	F	*p*
White blood cell (10^9^/L)	Time	2.462	0.064
	Group	0.254	0.859
Absolute value of lymphocytes (10^9^/L)	Time	0.501	0.682
	Group	0.196	0.899
CD3^+^ T cell (cells/μL)	Time	0.691	0.558
	Group	0.069	0.976
CD4^+^ T cell (cells/μL)	Time	0.968	0.409
	Group	0.171	0.916
CD8^+^ T cell (cells/μL)	Time	0.692	0.558
	Group	0.061	0.980
Hypersensitive C-reactive protein (mg/L)	Time	0.653	0.582
	Group	0.264	0.852
Interleukin-6 (pg/mL)	Time	1.033	0.355
	Group	0.212	0.792
Immunoglobulin G (g/L)	Time	1.241	0.297
	Group	0.891	0.447
Total protein (g/L)	Time	0.832	0.478
	Group	0.918	0.434
Albumin (g/L)	Time	0.164	0.921
	Group	0.858	0.464
Prealbumin (mg/L)	Time	0.448	0.719
	Group	0.448	0.719
Hemoglobin (g/L)	Time	0.775	0.510
	Group	0.956	0.415

**Table 3:** Comparison of perioperative intestinal barrier indicators between two groups and different times.

Variables	Statistical Indicators	F	*p*
Diamine oxidase (U/mL)	Time	11.364	0.000 ***
	Group	2.770	0.043 *
D-lactate (mg/L)	Time	20.263	0.000 ***
	Group	0.462	0.541
Endotoxin (EU/mL)	Time	2.844	0.039 *
	Group	2.772	0.043 *

* *p* < 0.05, *** *p* < 0.001.

## Data Availability

The raw sequence data reported in this paper have been deposited in the Genome Sequence Archive (Genomics, Proteomics & Bioinformatics 2021) in National Genomics Data Center (Nucleic Acids Res 2022), China National Center for Bioinformation/Beijing Institute of Genomics, Chinese Academy of Sciences (GSA: PRJCA044375) that are publicly accessible at https://ngdc.cncb.ac.cn/gsa.

## Supplementary Materials

The following supporting information can be downloaded at: https://www.mdpi.com/article/10.3390/biomedicines13092163/s1. Supplementary Figure S1. Genetic characteristics of each group. (a) Distribution of genes across groups. Color-coded bars represent the relative abundance of gene categories in PUIA, PUIB, PUNA, and PUNB. (b) Venn diagram illustrating the number of unique and shared differentially abundant genes between groups. Overlapping regions denote genes common to multiple groups. Group definitions: PUIA: preoperative standard nutrients. PUIB: postoperative standard nutrients. PUNA: preoperative immunonutrients group. PUNB: postoperative immunonutrients group. Supplementary Figure S2. VFDB analysis and CAZy analysis. (a) VFDB analysis between PUIA and PUIB. (b) CAZy analysis between PUIA and PUIB. Group definitions: PUIA: preoperative standard nutrients. PUIB: postoperative standard nutrients.
