# Supplementary material for: Perioperative Oral Immunonutrient Regulation of Intestinal Barrier and Gut Microbiota in Patients with Gastric Cancer, a Randomized Controlled Clinical Trial
Source: Biomedicines. 2025 Sep 5;13(9):2163. doi: 10.3390/biomedicines13092163 (PMC12467998; doi:10.3390/biomedicines13092163)
Supplement: Supplementary file 1 [file biomedicines-13-02163-s001.zip › biomedicines-3674403-Supplementary/Supplementary.pdf]

Supplementary Materials:

Supplementary Figure 1. Genetic characteristics of each group. (a) Distribution of genes across groups. Color-coded bars represent the relative abundance of gene categories in PUIA, PUIB, PUNA, and PUNB. (b) Venn diagram illustrating the number of unique and shared differentially abundant genes between groups. Overlapping regions denote genes common to multiple groups. Group definitions: PUIA: preoperative standard nutrients. PUIB: postoperative standard nutrients. PUNA: preoperative immunonutrients group. PUNB: postoperative immunonutrients group.

Supplementary Figure 2. VFDB analysis and CAZy analysis. (a) VFDB analysis between PUIA and PUIB. (b) CAZy analysis between PUIA and PUIB. Group definitions: PUIA: preoperative standard nutrients. PUIB: postoperative standard nutrients.
