# Supplementary figures and images for: Perioperative Oral Immunonutrient Regulation of Intestinal Barrier and Gut Microbiota in Patients with Gastric Cancer, a Randomized Controlled Clinical Trial
Source: Biomedicines. 2025 Sep 5;13(9):2163. doi: 10.3390/biomedicines13092163 (PMC12467998; doi:10.3390/biomedicines13092163)

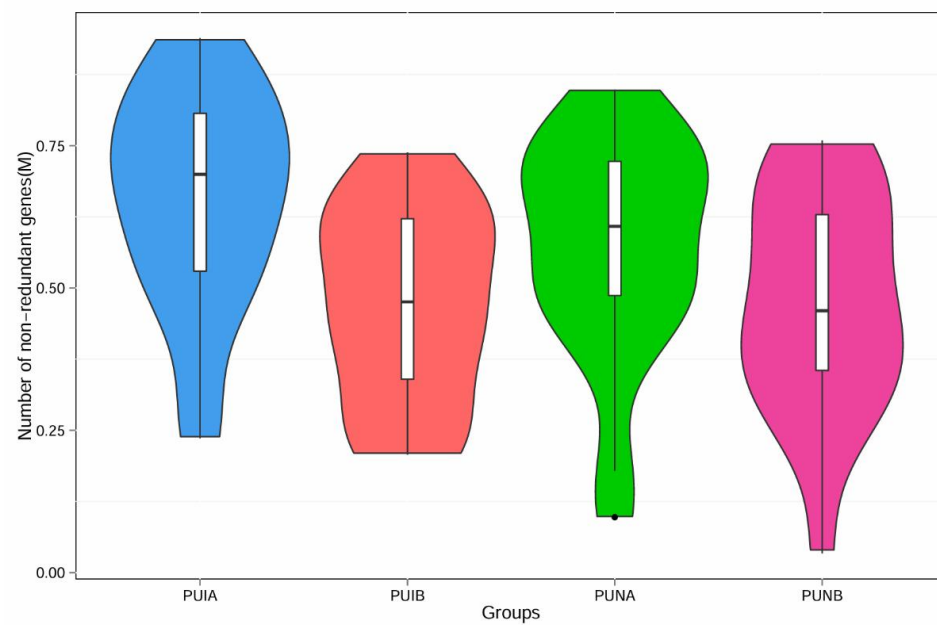

**(a)**

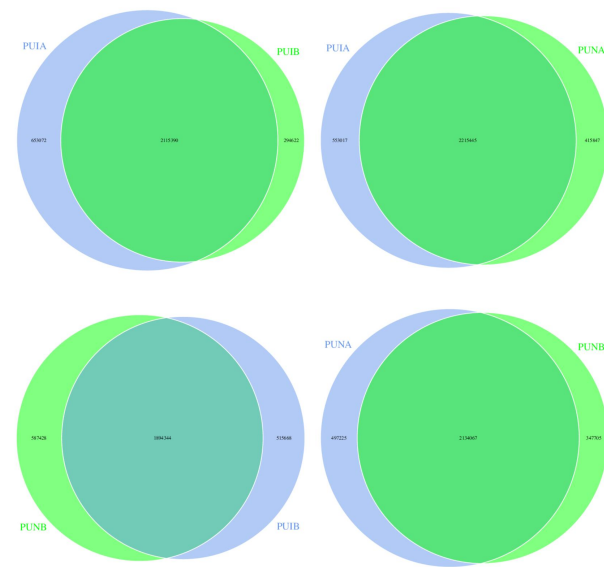

**(b)**

Supplement: Supplementary file 1 [file biomedicines-13-02163-s001.zip › biomedicines-3674403-Supplementary/Supplementary Figure 1 Genetic characteristics of each group.pdf]

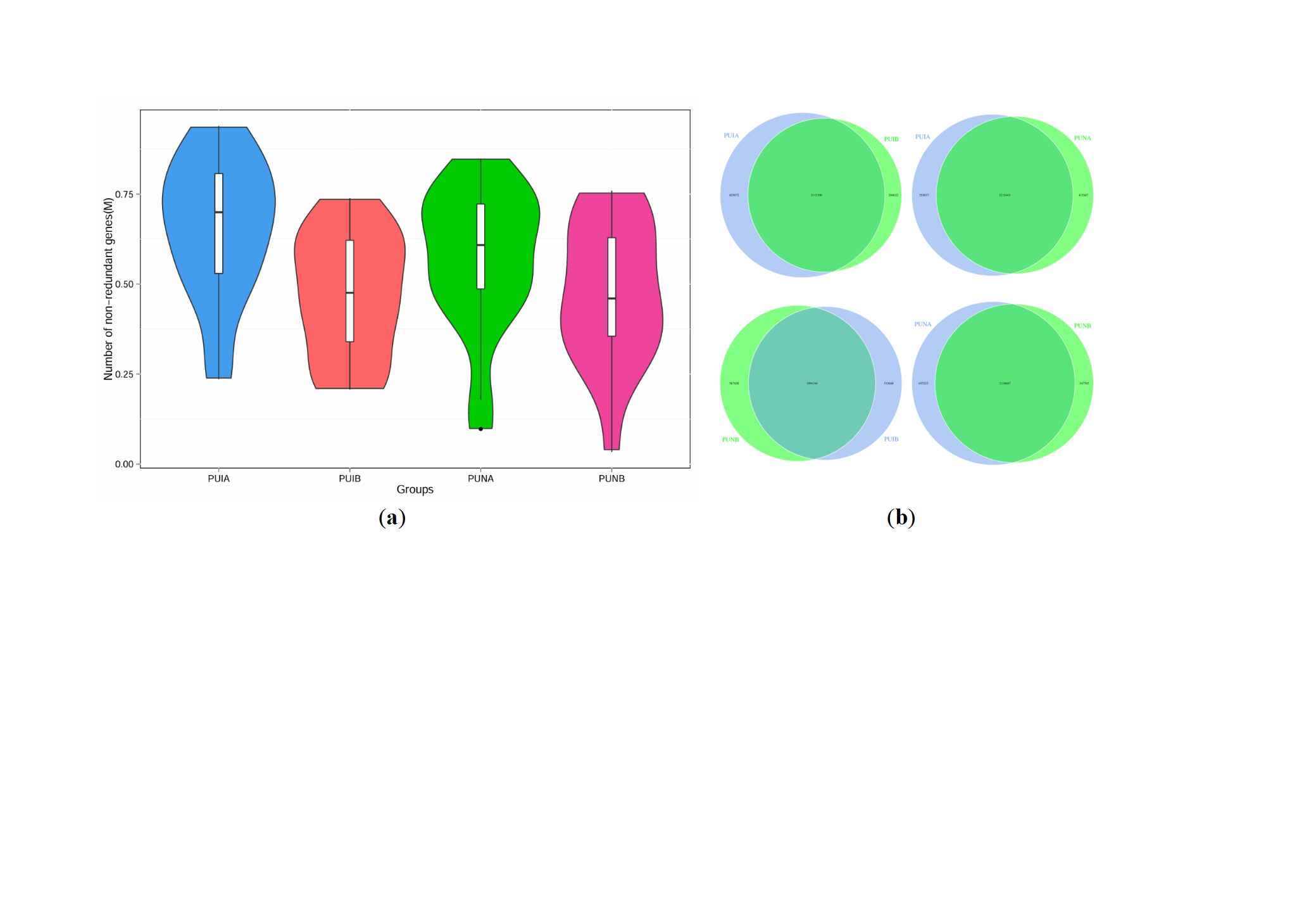

Supplement: Supplementary file 1 [file biomedicines-13-02163-s001.zip › biomedicines-3674403-Supplementary/Supplementary Figure 1 Genetic characteristics of each group.tif]

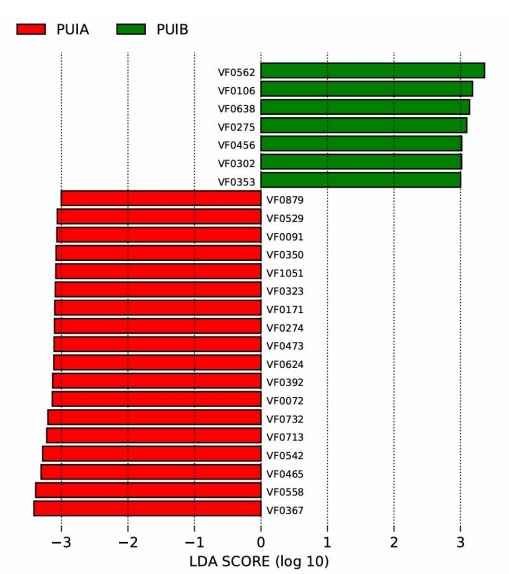

(a)

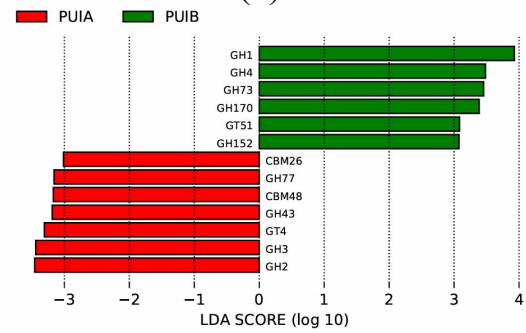

(b)

Supplement: Supplementary file 1 [file biomedicines-13-02163-s001.zip › biomedicines-3674403-Supplementary/Supplementary Figure 2 VFDB analysis and CAZy analysis.pdf]

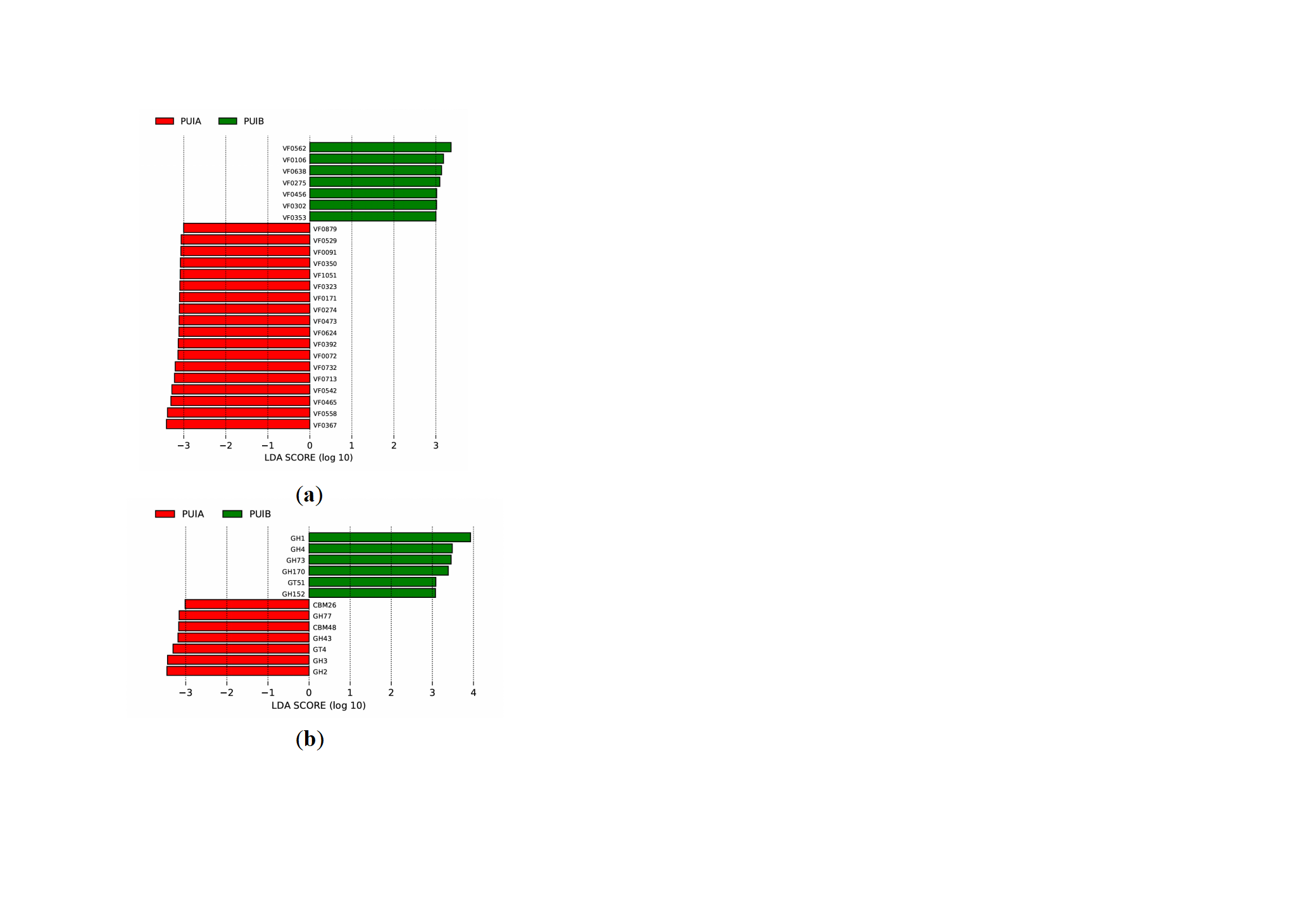

Supplement: Supplementary file 1 [file biomedicines-13-02163-s001.zip › biomedicines-3674403-Supplementary/Supplementary Figure 2 VFDB analysis and CAZy analysis.tif]
